# Does the Age Affect the Outcomes of Cardiac Resynchronization Therapy in Elderly Patients?

**DOI:** 10.3390/jcm10071451

**Published:** 2021-04-01

**Authors:** Teresa Strisciuglio, Giuseppe Stabile, Domenico Pecora, Giuseppe Arena, Salvatore Ivan Caico, Massimiliano Marini, Patrizia Pepi, Antonio D’Onofrio, Antonio De Simone, Giuseppe Ricciardi, Sandra Badolati, Alfredo Spotti, Gavino Casu, Francesco Solimene, Carmelo La Greca, Giuseppe Ammirati, Valerio Pergola, Lucio Addeo, Maurizio Malacrida, Emanuele Bertaglia, Antonio Rapacciuolo

**Affiliations:** 1 Department of Advanced Biomedical Sciences, Federico II University of Naples, 80138 Naples, Italy; teresa.strisciuglio@unina.it (T.S.); giuseppe.ammirati92@gmail.com (G.A.); valerio.pergola.pz@gmail.com (V.P.); addeolucio@gmail.com (L.A.); 2 Montevergine Clinic, 83013 Mercogliano, Italy; gmrstabile@tin.it (G.S.); francescosolimene@msn.com (F.S.); 3 Fondazione Poliambulanza, 25124 Brescia, Italy; domenico.pecora@poliambulanza.it (D.P.); carmelo.lagreca@poliambulanza.it (C.L.G.); 4 Department of Cardiology, Apuane Hospital, 54100 Massa, Italy; giuseppe.arena@uslnordovest.toscana.it; 5 Department of Cardiology, ASST Valle Olona, Gallarate Hospital, 21013 Gallarate, Italy; ivan.caico@asst-valleolona.it; 6 Department of Cardiology, Santa Chiara Hospital, 38122 Trento, Italy; massimiliano.marini@apss.tn.it; 7 Ospedale Carlo Poma, 46100 Mantua, Italy; patrizia.pepi@ASST-mantova.it; 8 Ospedale Monaldi, 80131 Naples, Italy; donofrioant1@gmail.com; 9 Clinica San Michele, 81024 Maddaloni, Italy; antodesimone2@alice.it; 10 Heart and Vessels Department, University of Florence, 50121 Firenze, Italy; giuseppe.ricciardi@unife.it; 11 Ospedale Sant’Andrea, 19121 La Spezia, Italy; sandra.badolati@asl5.liguria.it; 12 Istituti Ospitalieri, 26100 Cremona, Italy; alfredo.spotti@fastwebnet.it; 13 Department of Cardiology, Ospedale San Francesco, 08100 Nuoro, Italy; gavi.casu@tin.it; 14 Boston Scientific Italia, 20134 Milan, Italy; Maurizio.Malacrida@bsci.com; 15 Department of Cardiac, Thoracic, and Vascular Sciences, University of Padova, 35122 Padova, Italy; bertagliaferro@gmail.com

**Keywords:** cardiac resynchronization therapy, heart failure, elderly, clinical response, outcomes

## Abstract

Background: More and more heart failure (HF) patients aged ≥ 75 years undergo cardiac resynchronization therapy (CRT) device implantation, however the data regarding the outcomes and their predictors are scant. We investigated the mid- to long-term outcomes and their predictors in CRT patients aged ≥ 75 years. Methods: Patients in the Cardiac Resynchronization Therapy Modular (CRT MORE) Registry were divided into three age-groups: <65 (group A), 65–74 (group B) and ≥75 years (group C). Mortality, hospitalization, and composite event rate were evaluated at 1 year and during long-term follow-up. Results: Patients (*n* = 934) were distributed as follows: group A 242; group B 347; group C 345. On 12-month follow-up examination, 63% of patients ≥ 75 years displayed a positive clinical response. Mortality was significantly higher in patients ≥ 75 years than in the other two groups, although the rate of hospitalizations for HF worsening was similar to that of patients aged 65–74 (7 vs. 9.5%, respectively; *p* = 0.15). Independent predictors of death and of negative clinical response were age >80 years, chronic obstructive pulmonary disease (COPD) and chronic kidney disease (CKD). Over long-term follow-up (1020 days (IQR 680-1362)) mortality was higher in patients ≥ 75 years than in the other two groups. Hospitalization and composite event rates were similar in patients ≥ 75 years and those aged 65–74 (9 vs. 11.8%; *p* = 0.26, and 26.7 vs. 20.5%; *p* = 0.06). Conclusion: Positive clinical response and hospitalization rates do not differ between CRT recipients ≥ 75 years and those aged 65–74. However, age > 80 years, COPD and CKD are predictors of worse outcomes.

## 1. Introduction

Cardiac resynchronization therapy (CRT) is a validated strategy for improving cardiac pump function through biventricular pacing in heart failure (HF) patients with inter-ventricular conduction delay and mechanical dyssynchrony [1]. The incidence of HF increases with aging; indeed, a survey conducted by the European Society of Cardiology (ESC) revealed that, in current European practice, about 32% of CRT devices are implanted in patients aged ≥ 75 years [2]. However, as only a minority of patients included in the clinical trials belong to this age-group, whether CRT is still of benefit in these patients is debated. Previous studies have shown that CRT device implantation improves symptoms, quality of life and functional class in elderly people [3,4]. However, data regarding the outcomes and their predictors are scant and limited to old studies [5,6,7,8,9,10].

In this study, we analyzed the large database of the CRT MORE registry in order to investigate the clinical response, the mortality and the hospitalization rates in elderly CRT recipients (≥75 years).

## 2. Methods

### 2.1. Study Population

The Cardiac Resynchronization Therapy Modular (CRT MORE) Registry (clinicaltrials.gov Identifier: NCT01573091) was a prospective, single-arm, multi-center cohort study designed to evaluate the association between baseline and implantation variables and the outcomes of patients in whom a CRT device had been implanted in accordance with current guidelines [11]. Enrollment started in December 2011 and ended in November 2013 [12].

In the present analysis, the population of the CRT MORE registry was stratified into three groups according to age: <65 years (young, group A), between 65 and 74 (young old, group B), and ≥75 years (old, group C) [13], and comparisons were made among groups. The study complied with the Declaration of Helsinki, the local ethics committee approved the research protocol, and informed consent for data collection was obtained from the subjects.

### 2.2. CRT Implantation

In all patients enrolled in the Registry, devices and pacing leads were implanted by means of standard techniques and all devices were programmed in accordance with the clinical practice of each center. Procedural details have been previously described [12].

### 2.3. Clinical Response and Long-Term Outcomes

Clinical response was evaluated at 12 months; death from any cause and HF hospitalization, whichever occurred first after CRT implantation, were also evaluated. For the clinical evaluation, we used both the Clinical Response (CR) [14] and Clinical Composite Score (CCS) [15]. The CR was assessed in accordance with a hierarchical composite criterion comprising live status, hospitalization for HF, and variations in NYHA functional class. Specifically, a positive response was attributed to patients who remained alive without any episode of HF hospitalization after 12 months of CRT delivery and showed an improvement in NYHA class or remained in NYHA class I or II. A negative clinical response was attributed to patients who died or were hospitalized for signs of HF, showed worsening of their NYHA class or remained in NYHA class III or IV.

The CCS has been used as an intermediate-term primary endpoint in several trials of new interventions for the treatment of chronic heart failure. The CCS classifies each randomized patient as improved, unchanged, or worsened, according to the clinical response during the study and the clinical status at the end of the study. Patients are considered to have worsened if they have experienced a major clinical event or reported worsening of their NYHA class or global assessment.

Furthermore, left ventricle (LV) reverse remodeling was evaluated by measuring the effect of CRT on LV end-systolic volume (LVESV) and on left ventricle ejection fraction (LVEF), by comparing the baseline value with that recorded at the 12-month follow-up examination of surviving patients, and by calculating the proportion of patients who displayed a relative reduction of 15% or more in LVESV [14].

### 2.4. Statistical Analysis

Descriptive statistics are reported as means ± SD for normally distributed continuous variables, or medians with 25th to 75th percentiles in the case of skewed distribution. Normality of distribution was tested by means of the nonparametric Kolmogorov–Smirnov test. Differences between mean data were compared by means of a t-test for Gaussian variables, and the F-test was used to check the hypothesis of equality of variance. The Mann–Whitney non-parametric test was used to compare non-Gaussian variables. Differences in proportions were compared by applying χ^2^ analysis or Fisher’s exact test, as appropriate. Hazard ratios (HR) and their 95% confidence intervals (CI) were computed by means of a Cox regression model, in which baseline variables were fixed covariates and deaths or cardiovascular hospitalizations were time-dependent covariates.

The cumulative probability of death or HF hospitalization was displayed by means of the Kaplan–Meier method, and the log-rank test was used to compare cumulative events. A *p* value < 0.017 was considered significant after Bonferroni correction. All statistical analyses were performed by means of STATISTICA software, version 7.1 (Stat Soft, Inc., Tulsa, OK, USA).

### 2.5. Patient and Public Involvement Statement

Patients were not involved in the research study.

## 3. Results

### 3.1. Clinical Characteristics

According to age, 934 patients included in the CRT MORE registry were divided into three groups: 242 in group A (<65 years), 347 in group B (between 65 and 74 years), 345 in group C (≥75 years). Baseline clinical characteristics are reported in Table 1. In every group the majority were males and the mean age was, respectively: 80 ± 4, 70 ± 3, 57 ± 7; (A vs. C *p* < 0.0001; B vs. C *p* < 0.0001). The prevalence of patients in NYHA classes III–IV increased with age. Patients aged ≥75 years had more comorbidities (renal disease, atrial fibrillation, and hypertension) than those <65 years. In patients <65 years, an ischemic etiology of HF was more common (49.6% group A vs. 36% group C; *p* = 0.0014). The implantation of an implantable cardiac defibrillator (ICD) combined with a CRT decreased with age.

### 3.2. Clinical and Echocardiographic Response of Elderly Recipients of CRT Devices

At 12 months, the rate of positive CR was similar in patients aged 65–74 years and those ≥ 75 (68 vs. 63%; *p* = 0.18), whereas it was significantly higher in the youngest patient group (83.1%; *p* A vs. C = 0.0001; *p* A vs. B = 0.0001) (Table 2). On the other hand, 19.7% of patients aged 65–74 and 20% of patients aged ≥75 experienced worsening of CCS, which is almost twice the rate recorded in patients <65 years. By contrast, we did not find any difference among the groups in terms of echocardiographic response on 12-month follow-up examination (63.6% group A, 59.6% group B, 61.3%, group C; A vs. C *p* = 0.94; B vs. C *p* = 0.58).

### 3.3. One-Year and Long-Term Outcomes

Mortality at 12 months was higher in patients aged ≥75 years than in the other two groups, although the rate of hospitalization for worsening of HF was similar in patients ≥75 years and those aged 65–74.

**Figure 1:**
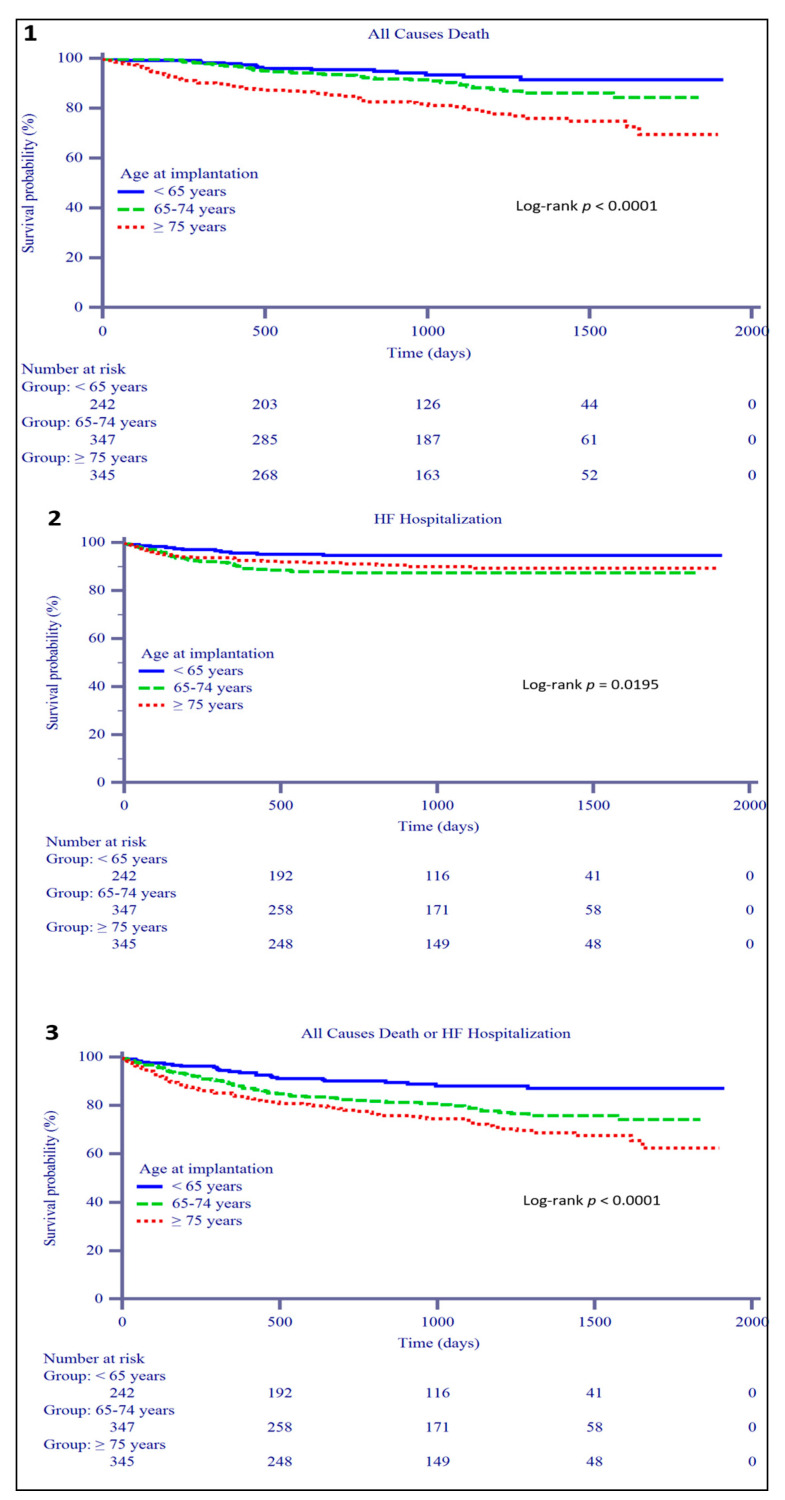
Long-term outcomes according to age group. The Kaplan–Meier curve for survival is shown in panel (**1**), whereas the Kaplan–Meier curves for heart failure (HF) hospitalization and composite events are shown in panels (**2**) and (**3**), respectively.

The 1-year composite event rate was 15.7% in patients ≥75 years, which was significantly higher only than that of patients aged <65 years (15.7% group C vs. 5.8% group A; *p* = 0.0002) (Table 3). Likewise, during a median follow-up of 1020 (680–1362) days, only the mortality rate was significantly higher in the oldest group, whereas hospitalization and composite event rates were similar to those of patients aged 65–74. (Figure 1).

There were no differences of outcomes in CRTD vs. CRTP patients in the three groups (Supplementary Table S1 and Supplementary Figure S1).

### 3.4. Predictors of Death and Association with CR and CCS at 1 Year in the Elderly Group (Age ≥ 75 Years)

**Figure 2:**
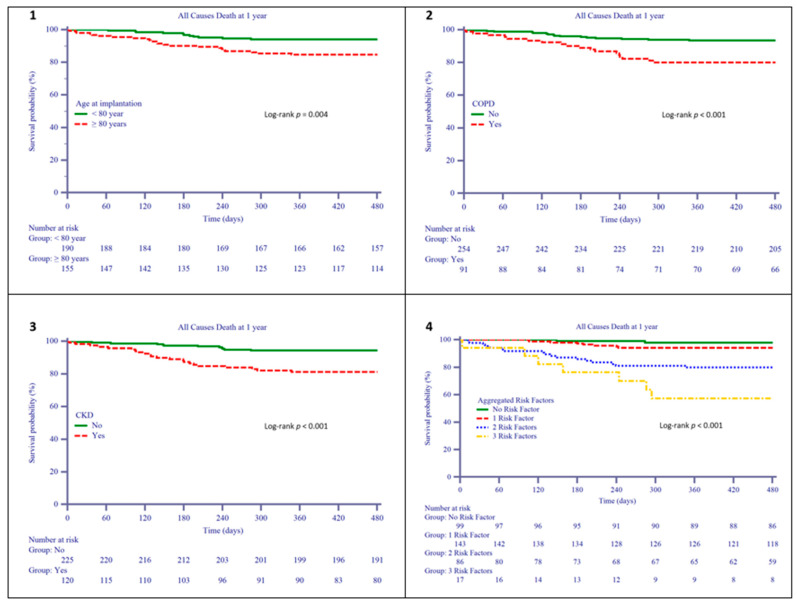
Predictors of death at 1 year in the elderly group. (**1**) All cause death in patients above and below 80 years old; (**2**) All cause death in patients with and without COPD; (**3**) All cause death in patients with and without CKD; (**4**) All cause death in patients with 1-2-3 or none of the abovementioned risk factors.The Kaplan–Meier curves show the reduction in survival in patients aged > 80 years (left upper panel), in patients with COPD (right upper panel) and in patients with CKD (left lower panel). Patients with all three factors have the lowest survival (right lower panel). COPD: chronic obstructive pulmonary disease; CKD: chronic kidney disease.

**Figure 3:**
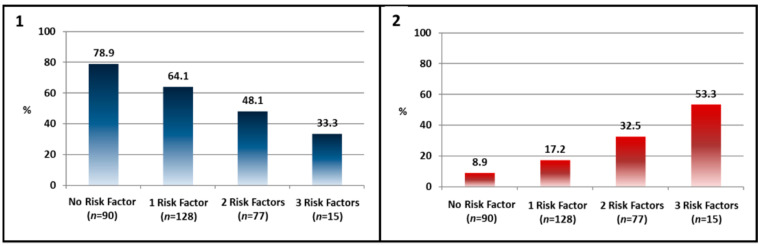
Positive Clinical Response and worsening of clinical composite score (CCS) according to the number of Risk Factors. As the number of risk factors increased, positive clinical response decreased (**1**) and rates of worsened CCS increased (**2**) (310 out of 345 cases available).

Death occurred more frequently in those aged >80 years, with atrial fibrillation (AF) at implantation, chronic obstructive pulmonary disease (COPD), chronic kidney disease (CKD), and in NYHA class III–IV (Table 4). On multivariate Cox regression analysis, adjusted for baseline confounders, only age >80 years, COPD and CKD ((Age: HR 2.32 (95% CI 1.1139–4.8319) *p* = 0.0253; COPD: HR 2.78 (95% CI 1.3763–5.6030) *p* = 0.0046; CKD: HR 2.70 (95% CI 1.3141–5.5463) *p* = 0.0071) remained associated with death. On plotting mean survival according to the number of risk factors, a clear separation of curves emerged between patients who had more than one predictor and those with 1 or no predictor (*p* < 0.0001) (Figure 2). Furthermore, these risk factors were also associated with the CCS and the CR; when these patients had more than 1 risk factor, the rate of positive CR progressively decreased, whereas the rate of patients with worsened CCS significantly increased (Figure 3).

## 4. Discussion

### 4.1. Main Findings

In the present study, we analyzed the clinical response, the mid- to long-term clinical outcomes and their predictors in a large elderly population included in the CRT MORE Registry. We found that: (1) at 1 year (mid-term follow-up), the rate of positive clinical response was similar in patients aged ≥ 75 years and in patients aged 65–74 years; likewise, the hospitalization and composite event rates were not significantly different; (2) age > 80 years, COPD and CKD were independent predictors of death at 1 year in elderly patients. The risk of death rose concomitantly with the presence of these factors, with the highest risk being observed in patients with all three factors; (3) these risk factors were also associated with a negative clinical response: patients with no risk factors had >80% probability of having a positive clinical response, whereas with two or more risk factors the probability was <30%; (4) over long-term follow-up, patients aged ≥ 75 years had similar HF hospitalization and composite event rates to patients aged 65–74, despite their higher mortality rate.

Our findings suggest that patients aged ≥ 75 years are good candidates for CRT, as the benefits are seen over both mid- and long-term follow-up. However, age > 80 years, CKD and COPD reduce the probability of positive clinical response and survival.

### 4.2. Elderly Patients and Medium—To Long-Term Clinical Outcomes after CRT Implantation

Nowadays, many people aged > 65 years are very active and, as suggested by Orimo et al., only patients aged 75 or above should be defined as “elderly” [16].

The European CRT survey provided important information on current European practice and revealed that about 32% of CRT devices are implanted in patients ≥ 75 years [2]. Whether these patients benefit from CRT has been investigated by previous studies, but their small sample sizes, short follow-up periods and differences in cut-off values used to categorize elderly patients have prevented this issue from being fully addressed.

Bleeker et al. [3] and Verbrugge et al. [4] reported that elderly recipients of CRT devices displayed similar improvements to those observed in younger patients in terms of clinical symptoms, NYHA class, quality-of-life scores and 6-min walking distance. Furthermore, no differences were found in the number of responders, the magnitude of LV ejection fraction improvement or the extent of LV remodeling.

In the large InSync/InSync ICD Italian registry, Fumagalli et al. [6] divided patients into three groups: <65 years, 65–74 and ≥75 years, as we did, and investigated their echocardiographic responses to CRT and long-term outcomes. However, these patients were enrolled between 1999 and 2005; since then, many improvements in CRT have been made and also the echocardiographic cut-off used to define responders has changed. Therefore, new data on medium- and long-term outcomes were needed.

The CRT MORE Registry holds data on patients who underwent CRT implantation from 2011 to 2013. Although previous studies have analyzed outcome and its predictors in this large population [14,17,18], these outcomes have never been analyzed in relation to age.

In the present sub-study, we found that patients aged ≥ 75, despite their higher mortality, had a similar rate of positive clinical response at 1 year to that of patients aged 65–74 (63 and 68%, respectively). As expected, on long-term follow-up their mortality remained higher, although their hospitalization rate was similar. Interestingly, however, age ≥ 80 years, like CKD and COPD, identified elderly patients with a lower probability of a positive clinical response and a higher rate of death.

### 4.3. Clinical Perspectives

In the last decades, patients affected from cardiovascular diseases have greatly improved their prognosis especially in terms of mortality rate reduction. For instance, people with acute myocardial infarction very often survive thank to a wide use of early invasive coronary revascularization. As a consequence, along with the increasing age of the population, heart failure patients are continuously increasing.

We currently have several pharmacological and invasive strategies to ameliorate the clinical status, as well as the prognosis of these patients. Therefore, it is becoming always more important to better identify the right patient needing a specific procedure.

Our findings have important clinical implications, as they demonstrate that elderly patients can still benefit from CRT device implantation. However, the presence of comorbidities such as CKD and COPD, especially in those aged >80, should be a warning sign as these conditions may synergistically interact and reduce the benefits of this approach.

The VALID-CRT prognostic score has recently been demonstrated to predict both mortality and clinical response [14]. In this score, however, of the three aforementioned predictors, only age is taken into account. We therefore identified two novel variables for risk stratification and for tailored treatment.

As for the influence of the ICD on outcomes, we found no difference between CRTP and CRTD patients. However, the goal of our study was to understand clinical response to CRT in the elderly, therefore a specific evaluation of the difference of CRTP vs. CRTD cannot be done in our population. We believe that only a randomized trial would address this question. In fact, CRTP is usually implanted in patients with more comorbidities and without a RCT study this would create a very significant bias in the results.

Current ESC guidelines recommend implanting a CRT-D if life-expectancy is >1 year [11]. However, as the procedure is associated with more complications, a longer in-hospital stay and a higher risk of infections, it may prove cost-effective only in patients who are expected to live 5–7 years after implantation [19]. As the prevalence of CKD and COPD is especially high in octogenarians, therefore every physician when implanting a CRT should be aware that the probability of clinical improvement in these patients is lower.

## 5. Limitations

(1) The data used in this study were taken from a registry; we cannot therefore exclude the presence of some selection bias;

(2) As the CRT MORE is a multicenter registry, we cannot guarantee that data collection was homogenous, although all centers followed a pre-specified protocol;

(3) We did not assess the frailty of the patients. Thus, it is possible that the results would have been different in a very frail population;

(4) Pharmacological therapy on enrollment was not optimal, especially in terms of β-blockers and angiotensin-converting enzyme inhibitors, as treatment was based on clinical evaluation by the attending physicians. However, this observational prospective study may provide a representative image of the real-life scenario of pharmacological therapy in patients undergoing CRT implantation;

(5) The echocardiographic data at follow-up were available only for 589 (63%) patients, therefore the ad hoc analysis was not performed.

## 6. Conclusions

Elderly patients ≥ 75 years still have benefit from the CRT implantation as similar rates of positive clinical response are seen in patients ≥ 75 years and those aged 65–74. Although elderly patients have higher mortality, however this is driven by the age itself, as conversely the hospitalizations rates do not differ from those of patients aged 65–74.

Predictors of worse outcomes are age > 80 years, CKD and COPD. A proper characterization of baseline parameters can be helpful to estimate upfront the probability of response to the CRT.

## Figures and Tables

**Table 1:** Baseline characteristics and Indication Class.

Parameter	All (*n* = 934)	<65 years (*n* = 242) (A)	65–74 years (*n* = 347) (B)	≥75 years (*n* = 345) (C)	*p* * C vs. A	*p* * C vs. B
Age, years	70 ± 10	57 ± 7	70 ± 3	80 ± 4	<0.0001	<0.0001
Gender Male	687 (73.6)	185 (76.4)	260 (74.9)	242 (70.1)	0.0232	0.0375
NYHA Class: III/IV	567 (60.7)	131 (54.1)	197 (56.8)	239 (69.3)	0.0003	0.0009
Ischemic	433 (46.4)	87 (36)	175 (50.4)	171 (49.6)	0.0014	0.8791
CKD	237 (25.4)	31 (12.8)	86 (24.8)	120 (34.8)	<0.0001	0.0052
COPD	225 (24.1)	47 (19.4)	87 (25.1)	91 (26.4)	0.0633	0.7599
GFR	62 (43–85)	87 (70–112)	67 (47–83)	45 (35–61)	<0.0001	<0.0001
Diabetes	290 (31.0)	74 (30.6)	125 (36)	91 (26.4)	0.3074	0.0079
Hypertension	607 (65.0)	131 (54.1)	233 (67.1)	243 (70.4)	0.0001	0.3946
Persistent/Permanent AF	198 (21.2)	36 (14.8)	75 (21.6)	87 (25.2)	0.0034	0.3032
QRS duration, ms	155 ± 26	153 ± 25	155 ± 25	156 ± 28	0.1272	0.4933
PR duration, ms	189 ± 49	184 ± 41	184 ± 44	200 ± 57	0.0021	0.0006
LBBB	763 (81.4)	195 (80.6)	288 (83)	280 (81.2)	0.9445	0.5963
MR ≥ 2	454 (48.6)	105 (43.4)	174 (50.1)	175 (50.7)	0.0954	0.9389
LVEF, %	29 ± 7	28 ± 8	29 ± 7	30 ± 7	0.0131	0.0345
CRTD	820 (87.8)	231 (95.5)	321 (92.5)	268 (77.7)	<0.0001	<0.0001
RV Apex	631 (66.6)	164 (67.8)	233 (67.1)	234 (67.8)	0.9402	0.9128
Diuretics	796 (85.2)	206 (85.1)	296 (85.3)	294 (85.2)	0.931	0.9397
ACE/ARB	688 (73.7)	200 (82.6)	241 (69.5)	247 (71.6)	0.0028	0.593
Anti-arrhythmics	212 (22.7)	53 (21.9)	79 (22.8)	80 (23.2)	0.7897	0.9669
Statin	426 (45.6)	97 (40.1)	171 (49.3)	158 (45.8)	0.1969	0.4003
BB	750 (80.3)	207 (85.5)	279 (80.4)	264 (76.5)	0.0095	0.2503

* *p* < 0.017 was considered significant after Bonferroni correction. NYHA is New York Heart Association; CKD is chronic kidney disease; COPD is Chronic obstructive pulmonary disease; GFR is glomerular filtration rate; AF is atrial fibrillation; LBBB is left bundle branch block; MR is mitral regurgitation; LVEF is left ventricle ejection fraction; CRTD is cardiac resynchronization therapy; RV is right ventricle; ACE/ARB is Angiotensin converting enzyme inhibitors/Angiotensin II Receptor Blockers; BB is beta-blocker.

**Table 2:** Clinical Response and Clinical Composite Score at 1 year.

At 1-Year Follow-Up Examination	All Pts (*n* = 836)	Age < 65 (*n* = 207) (A)	Age 65–74 (*n* = 319) (B)	Age ≥ 75 (*n* = 310) (C)	*p* A vs. B	*p* C vs. A	*p* C vs. B
Positive Clinical Response, *n* (%)	584 (69.9)	172 (83.1)	217 (68.0)	195 (62.9)	0.0001	0.0001	0.1807
Worsened Status on CCS, *n* (%)	146 (17.5)	21 (10.1)	63 (19.7)	62 (20.0)	0.0034	0.0032	1.0

**Table 3:** Medium-term outcomes.

At 1 Year Follow Up	All Pts (*n* = 934)	Age < 65 (*n* = 242)(A)	Age 65–74 (*n* = 347)(B)	Age ≥ 75 (*n* = 345)(C)	*p* A vs. B	*p* C vs. A	*p* C vs. B
Overall Death, *n* (%)	47 (5.0)	4 (1.7)	9 (2.6)	34 (9.9)	0.5736	<0.0001	<0.0001
HF Hospitalization, *n* (%)	67 (7.2)	10 (4.1)	33 (9.5)	24 (7.0)	0.0152	0.2083	0.2687
Death or HF Hospitalization, *n* (%)	108 (11.6)	14 (5.8)	40 (11.5)	54 (15.7)	0.0197	0.0002	0.1488

**Table 4:** Predictors of death at 1 year in the elderly group.

	Univariate			Multivariate		
Parameter	HR	95% CI	*p*	HR	95% CI	*p*
Persistent/Permanent AF on implantation	2.1081	1.0447 to 4.2540	0.0383	1.73	0.8492 to 3.5246	0.1331
Age ≥ 80 years	2.7145	1.3281 to 5.5484	0.0065	2.32	1.1139 to 4.8319	0.0253
BMI	0.9873	0.9063 to 1.0756	0.7712			
COPD	3.2514	1.6636 to 6.3546	0.0006	2.78	1.3763 to 5.6030	0.0046
Diabetes	0.7321	0.3191 to 1.6794	0.4639			
Echo MR ≥ 2	2.0979	0.9918 to 4.4377	0.0538			
Gender Male	1.1317	0.5537 to 2.3132	0.7358			
CKD	3.6942	1.8345 to 7.4391	0.0003	2.70	1.3141 to 5.5463	0.0071
Hypertension	1.345	0.6114 to 2.9587	0.4636			
CRT-D	1.1171	0.4886 to 2.5542	0.794			
Ischemic	1.2627	0.6439 to 2.4765	0.4994			
LBBB	1.3702	0.5330 to 3.5225	0.5154			
QRS	0.998	0.9860 to 1.0101	0.7463			
NYHA III/IV vs. I/II	4.8602	1.4948 to 15.8026	0.0089	2.67	0.7979 to 8.9577	0.1128
LVEF on implantation	0.9732	0.9268 to 1.0219	0.2782			
ESC 2016 Indication Class	1.4935	0.9275 to 2.4049	0.1006			

BMI is body mass index; COPD is chronic obstructive pulmonary disease; MR is mitral regurgitation; CKD is chronic kidney disease; CRT-D is cardiac resynchronization therapy-defibrillator; LBBB is left bundle branch block; QRS is QRS width; NYHA is New York Heart Association; LVEF is left ventricle ejection fraction.

## Data Availability

Not applicable.

## Supplementary Materials

The following are available online at https://www.mdpi.com/article/10.3390/jcm10071451/s1, Table S1: Outcomes in CRTD vs. CRTP patients in the three groups and Figure S1: Kaplan-Meier curves of CRTD vs. CRTP patients.
