# Supplementary material for: Does the Age Affect the Outcomes of Cardiac Resynchronization Therapy in Elderly Patients?
Source: J Clin Med. 2021 Apr 1;10(7):1451. doi: 10.3390/jcm10071451 (PMC8036418; doi:10.3390/jcm10071451)
Supplement: Supplementary file 1 [file jcm-10-01451-s001.zip › Supplementary table 1.pdf]

Supplementary table 1. Outcomes in CRTD vs CRTP patients in the three groups

At 1-year follow-up examination	All pts (n=934)	All pts CRT-D (n=820)	All pts CRT-P (n=114)	Age <65 (n=242) (A)	Age <65 CRT-D (n=231) (A1)	Age <65 CRT-P (n=11) (A2)	Age 65-74 (n=347) (B)	Age 65-74 CRT-D (n=321) (B1)	Age 65-74 CRT-P (n=26) (B2)	Age ≥75 (n=345) (C)	Age ≥75 CRT-D (n=268) (C1)	Age ≥75 CRT-P (n=77) (C2)
**Overall Death, n (%)**	47 (5.0)	40 (4.9)	7 (6.1)	4 (1.7)	4 (1.7)	0 (0.0)	9 (2.6)	9 (2.8)	0 (0.0)	34 (9.9)	27 (10.1)	7 (9.1)
**HF Hospitalization, n (%)**	67 (7.2)	61 (7.4)	6 (5.3)	10 (4.1)	10 (4.3)	0 (0.0)	33 (9.5)	30 (9.3)	3 (11.5)	24 (7.0)	21 (7.8)	3 (3.9)
**Death or HF Hospitalization, n (%)**	108 (11.6)	95 (11.6)	13 (11.4)	14 (5.8)	14 (6.1)	0 (0.0)	40 (11.5)	37 (11.5)	3 (11.5)	54 (15.7)	44 (16.4)	10 (13.0)
