# Supplementary figures and images for: Does the Age Affect the Outcomes of Cardiac Resynchronization Therapy in Elderly Patients?
Source: J Clin Med. 2021 Apr 1;10(7):1451. doi: 10.3390/jcm10071451 (PMC8036418; doi:10.3390/jcm10071451)

Supplementary figure 1. Kaplan-Meier curves of CRTD vs CRTP patients.

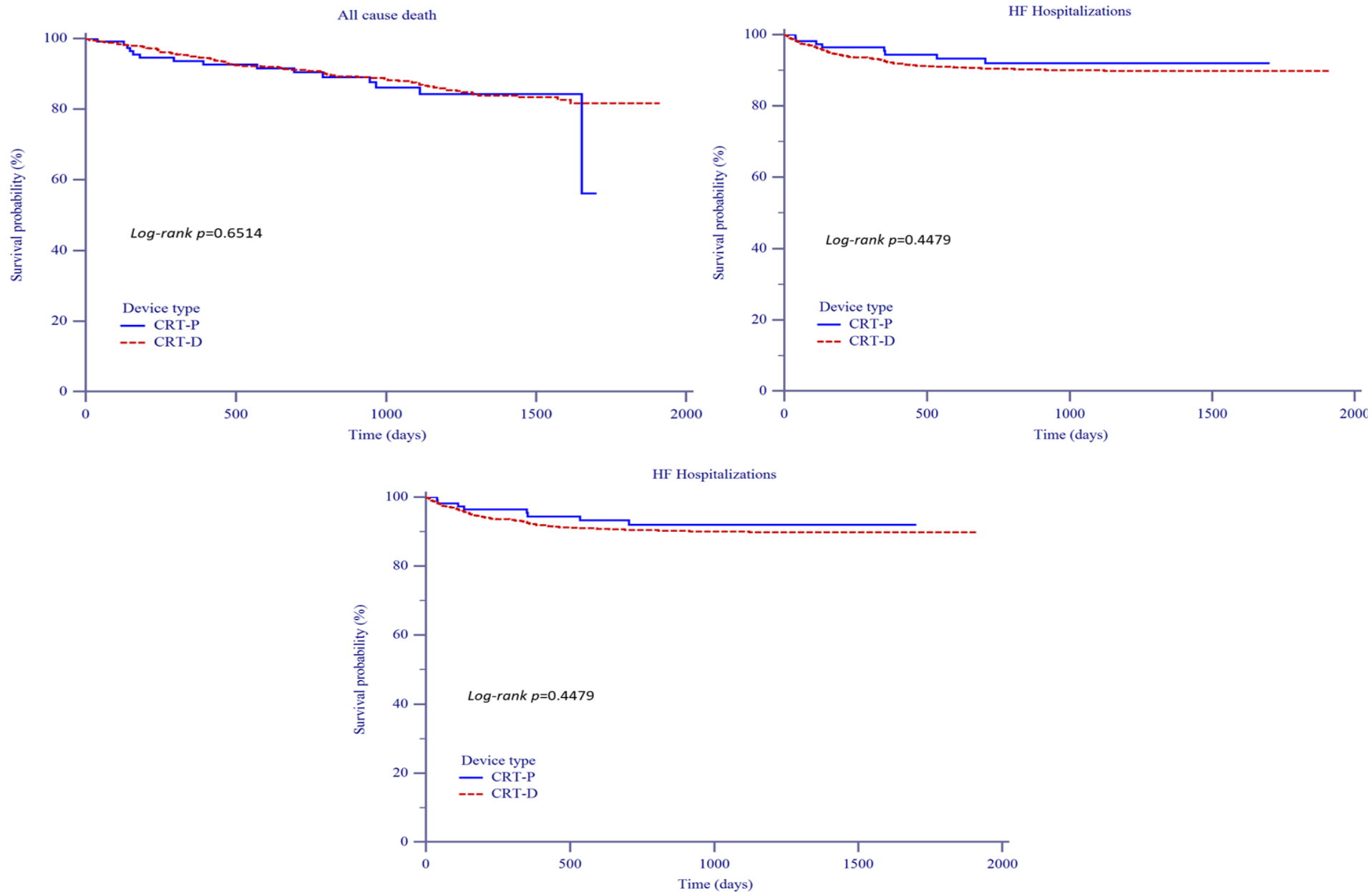

Supplement: Supplementary file 1 [file jcm-10-01451-s001.zip › Supplementary figure 1.pdf]
